# Olfactory Function and Diffusion Tensor Imaging as Markers of Mild Cognitive Impairment in Early Stages of Parkinson's Disease

**DOI:** 10.1177/15500594211058263

**Published:** 2021-11-29

**Authors:** Sophie A. Stewart, Laura Pimer, John D. Fisk, Benjamin Rusak, Ron A. Leslie, Gail Eskes, Kerrie Schoffer, J. Roger McKelvey, Tyler Rolheiser, M. Naeem Khan, Harold Robertson, Kimberley P. Good

**Affiliations:** 1 3688 Dalhousie University, Halifax, NS, Canada; 2 432234 Nova Scotia Health, Halifax, NS, Canada; 3 3682 IWK Hospital, Halifax, NS, Canada

**Keywords:** Parkinson's disease, mild cognitive impairment, olfaction, diffusion tensor imaging

## Abstract

Parkinson's disease (PD) is a neurodegenerative disorder that is typified by motor signs and symptoms but can also lead to significant cognitive impairment and dementia Parkinson's Disease Dementia (PDD). While dementia is considered a nonmotor feature of PD that typically occurs later, individuals with PD may experience mild cognitive impairment (PD-MCI) earlier in the disease course. Olfactory deficit (OD) is considered another nonmotor symptom of PD and often presents even before the motor signs and diagnosis of PD. We examined potential links among cognitive impairment, olfactory functioning, and white matter integrity of olfactory brain regions in persons with early-stage PD. Cognitive tests were used to establish groups with PD-MCI and with normal cognition (PD-NC). Olfactory functioning was examined using the University of Pennsylvania Smell Identification Test (UPSIT) while the white matter integrity of the anterior olfactory structures (AOS) was examined using magnetic resonance imaging (MRI) diffusion tensor imaging (DTI) analysis. Those with PD-MCI demonstrated poorer olfactory functioning and abnormalities based on all DTI parameters in the AOS, relative to PD-NC individuals. OD and microstructural changes in the AOS of individuals with PD may serve as additional biological markers of PD-MCI.

PD is the second most common neurodegenerative disorder after Alzheimer's disease^1^ and is characterized by a distinct set of motor and nonmotor symptoms. Classic motor symptoms of PD include a “pill rolling” tremor, bradykinesia (slowness of movement), muscular rigidity, and balance problems leading to postural instability.^2^ Nonmotor symptoms of PD which often predate the onset of frank motor deficits, include depression, sleep disorders, constipation, olfactory impairment, and cognitive deficits.^2^

Pathological studies have suggested that PD develops in 6 stages over a period of several decades.^3^ While pathology in the anterior olfactory structures (AOSs) is thought to occur in stages 1 or 2, diagnosis does not typically take place until stage 3, when motor signs and symptoms become apparent. At that time, Lewy Bodies are found in the midbrain, basal forebrain, and substantia nigra. Well into the disease course, during stages 5 and 6, it is thought that abnormal aggregation of alpha-synuclein proteins is widespread throughout the entire cortex.^3^ It is during these later 2 stages that dementia is typically observed.

While as many as 80% of PD patients may develop dementia (PDD) over the course of their illness,⁴ mild cognitive impairment has been reported in 15% to 43% of people newly diagnosed with PD.⁵ Standardized guidelines for the diagnosis of PD-MCI persons were developed by the Movement Disorder Society (MDS) Task Force and published in 2012;⁶ these guidelines reflect an intermediate state that falls on the spectrum between normal cognition and dementia. Subsequent studies using these criteria have demonstrated an elevated risk of developing dementia in those with PD-Mild cognitive impairment (MCI)^6-8^ though PD-MCI cannot be considered predictive of future PDD over the medium term of up to 5 years. Nevertheless, over longer follow up periods, the risk of PDD for those with PD-MCI has been estimated at up to 91%.⁹ Some proportion of those with PD-MCI remain stable or even revert to normal cognition.^5,7,9^ However, even those who are found to normalize may still be at an increased risk of future development of dementia relative to those who have never shown signs of cognitive impairment.^10^ Demographic factors, comorbidities, and patterns of cognitive test performance have been shown to have some associations with the risk of development of PDD but associations between PD-MCI, and other nonmotor symptoms and biomarkers have the potential to further clarify this risk.

One of the hallmarks and earliest presenting nonmotor symptoms of PD is OD. OD is experienced by 90% to 95% of people with PD, when compared to age-matched controls.^11,12^ Olfactory deficit (OD) usually presents itself before the onset of motor symptoms by about 4 to 6 years.^13^ The exact pathology of OD in PD is unclear, but it is likely related to changes in the AOSs, such as the olfactory bulbs and olfactory tracts.^14^ Studies of OD in PD have revealed that poorer olfaction is associated with greater cognitive decline. PD patients with greater OD were more likely to develop MCI than PD patients with less severe or no OD.^15^ In prior MRI studies using DTI, Rolheiser et al^16^ and Joshi et al^17^ demonstrated microstructural abnormalities in AOSs (olfactory bulbs and tracts) in patients with PD that are related to the presence of OD. Thus, the presence of OD together with MRI evidence of degeneration of olfactory regions in early stages of PD might allow detection of higher risk of developing more severe or rapid cognitive decline.

In the current study, we examined a group of patients with a confirmed diagnosis of early PD and compared those who met criteria for PD-MCI to those with PD-NC on measures of olfactory function and DTI measures of the olfactory bulbs and tracts. We aimed to determine whether olfaction and white matter microstructural abnormalities differed between those with PD-MCI and those with PD-NC. We hypothesized that persons with PD-MCI would display lower olfactory identification abilities and would show greater microstructural abnormalities than persons with PD-NC as assessed using DTI metrics of their olfactory bulbs and tracts.

## Methods

### Subjects

Participants (19 male and 14 female) were recruited from the Movement Disorder Clinic of the Division of Neurology at Nova Scotia Health, Halifax Canada. All participants had been diagnosed by a movement disorder specialist (K.S. and J.R.M.) as having PD and rated less than 3 on the Hoehn & Yahr scale.^18^ In order to be included in this study participants had to have normal/corrected-to-normal vision and hearing, no contraindications for MRI scanning, no other explanations for cognitive impairment (eg, stroke, head trauma), and no PD-associated comorbid conditions that could affect cognition (eg, severe anxiety, depression). Participants averaged 62 ± 6.4 (SD) years of age (range: 46-70 years). The mean number of years of school completed was 14.8 years (*SD* = 3.8). The Nova Scotia Health Authority Research Ethics Board approved the study.

### Materials

#### Cognitive Tests

A battery of cognitive tests was used to establish whether participants qualified as having normal cognition or MCI. For each test, raw test scores were converted to z-scores based on the age-adjusted published normative data on healthy individuals.^a^ The neuropsychological tests examined the 5 cognitive domains included in the MDS guidelines for PD-MCI.⁶

The cognitive test battery included the following domains: 1) visuospatial/constructional ability (Wechsler Abbreviated Scale of Intelligence [WASI] *Block Design*^19^); 2) episodic verbal memory (The California Verbal Learning Test [CVLT]^20^); 3) speed of processing (The Symbol Digit Modalities Test [SDMT]^21^); 4) executive functioning (The D-KEFS Verbal Fluency Test [D-KEFS VF]^22^) and (Delis Kaplan Executive Function System [D-KEFS] Trail Making Test [D-KEFS TMT]^22^); 5) visual working memor**y** (wechsler memory scale [WMS-III *Spatial Span*^23^).

#### MCI Assessment

We employed the MDS level I/abbreviated criteria for PD-MCI (6) with slight adaptation. To address the lack of specificity in the definition of “impairment” in the abbreviated assessment, a cutoff score of 1.5 SD below the appropriate normative data was added to our PD-MCI criteria. A cutoff score of 1.5 SD below the norm for defining MCI has been used widely in the literature (eg,^24,25^). For the purpose of this study, a participant who demonstrated impairment on any 2 tests was considered PD-MCI.

##### Olfactory Assessment

All subjects underwent an assessment of olfactory functioning using the UPSIT. The UPSIT is a 40-item test that involves participants scratching a patch that includes a microencapsulated scent.^26^ The participant sniffs the scent patch and makes a decision as to what the odorant is (eg, pizza, spearmint) from 4 options. This is a forced choice task in that every item requires a response even if no apparent odor is detected. Performance is based on the number of correctly identified odors, with each correct answer carrying a score of one point (of 40).^27^ Extensive normative data suggests that any score above 33 is “normal” and for individuals in the 60 to 70 year range, scores between 10 to 20 are typical for “total anosmics” (Doty et al^28^). The UPSIT has high reliability for smell identification (r = 0.94) and is simple to administer.^29^

#### Diffusion Tensor Imaging

As described previously,^16^ all patients underwent MRI with DTI to assess the integrity of and potential alterations in, white matter microstructure in the brain. A GE Signa HDxt 1.5 T whole body magnet with an 8HRBrain coil located in the IWK Health Center, Halifax, NS, Canada, was used to obtain MRI images, including T1-weighted, T2-weighted, and diffusion weighted images. Images were preprocessed with a computer brain imaging data analysis software called FSL,^30^ which included skull stripping, linear and nonlinear registration, and eddy current correction. Region of interests (ROIs) were traced and an analysis was carried out to calculate tensor fractional anisotropy (FA), axial diffusivity (AD), radial diffusivity (RD), and mean diffusivity (MD). The ROIs selected for analysis were the left and right AOSs which included the olfactory bulbs and tracts. Tracing was done blind to subgroup membership and to olfactory performance.

## Procedure

As part of a larger study, UPSIT scores, MRI, and cognitive tests scores were obtained from all participants. Informed consent was obtained, a demographic questionnaire was completed, and cognitive tests, including the UPSIT, were administered. The MRI scan took place on a separate day (typically within one week of the first visit). If the patient preferred to complete the cognitive testing in 2 sessions (because the participant became fatigued or the examiner felt that the participant was too tired to perform appropriately), a third visit was planned.

### Statistical Analyses

Based on cognitive testing, patients were divided into PD-MCI and PD-NC groups. Independent sample t-tests were used to compare group scores on all cognitive tests. An independent sample t-test was also used to compare the PD-MCI and PD-NC groups on UPSIT scores. Finally, a 2-way mixed-design analysis of variance compared group scores on all DTI parameters. A within-subject factor (Side) included the right and left AOS regions (ie, olfactory tracts and olfactory bulbs) while a between-group factor (Group) compared the participants. The significance level for all tests was set a *P* <.05 and Jamovi (version 1.2.27) was used for all statistical analyses.

## Results

Using the scaled scores from the CVLT (recall trials 1-5), we identified 12 subjects who met criteria for PD-MCI and 21 who were classified as PD-NC. These groups did not differ on the basis of age, sex ratio, or years of education. Both groups were equally impaired according to the Hoehn & Yahr ratings, nor did they differ in duration of PD diagnosis or years since symptom onset. The Unified Parkinson Disease Rating Scale (UPDRS) is a clinician-rated scale that is used to gauge the progression of motor impairment (Part III: range 0—no impairment to 56—extremely impaired) in patients; groups did not differ in UPDRS scores. For more information on the groups, see Table 1.

**Table 1.** Demographics and Disease-Related Variables of PD-MCI and PD-NC Groups.

	PD-MCI (n = 12)		PD-NC (n = 21)			
Demographic and disease-related variables	M	SD	M	SD	Student's t	*P*
Age (y)	63.5	7.28	61.4	5.95	−0.89	.38
Sex (male/female)	6/6	0.52	13/8	0.50	−0.65	.52
Education	13.6	2.94	15.5	4.15	1.39	.17
Hoehn & Yahr	1.88	0.53	1.71	0.60	−0.77	.45
UPDRS	24.0	5.02	21.6	12.8	1.08	.29
Duration from diagnosis (y)	4.42	3.95	2.43	2.64	−1.74	.09
Duration from symptom onset (y)	4.36	3.21	3.89	2.99	−0.41	.69

Abbreviations: PD-MCI, Parkinson's disease with mild cognitive impairment; PD-NC, Parkinson's disease with normal cognition; UPDRS, Unified Parkinson Disease Rating Scale.

**Figure 1.**
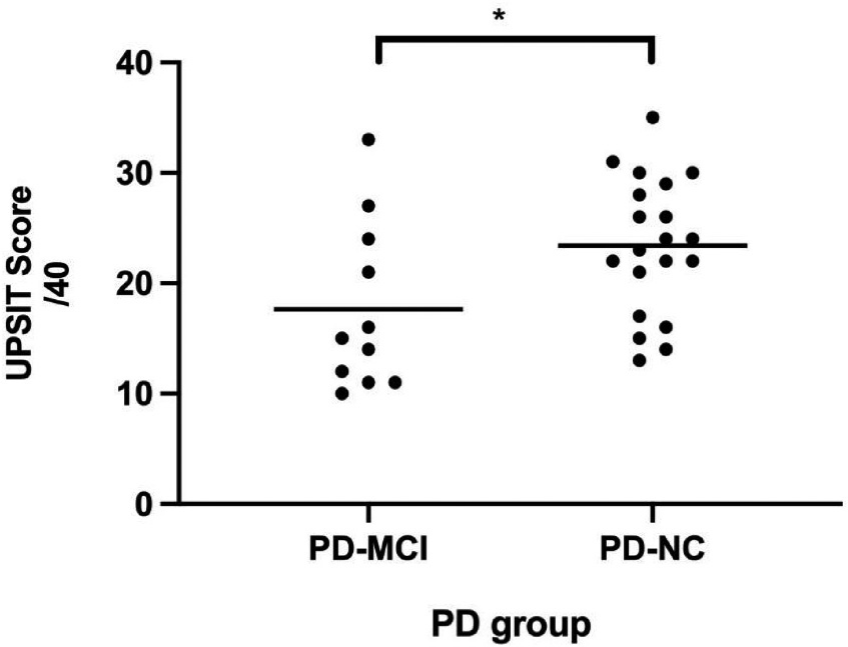
Mean UPSIT scores (of 40) of PD-MCI and PD-NC groups. Each individual subject is represented by a dot and each group mean is represented by a horizontal bar. *P <.05.

Abbreviations: PD-MCI, Parkinson's disease with mild cognitive impairment; PD-NC, Parkinson's disease with normal cognition; UPSIT, University of Pennsylvania Smell Identification Test.

The PD-MCI and PD-NC groups differed on scores from the CVLT, D-KEFS TMT (ie, numbers, switching, motor, scanning), and SDMT but not the WASI Block Design, D-KEFS VF, or WMS III Spatial Span (Table 2). UPSIT data were missing for 2 subjects (one from each of the 2 groups). UPSIT scores for the PD-MCI group (mean = 17.6; *SD* *=* 7.59) differed from those of the PD-NC group (mean = 23.4; *SD* = 6.16) (t[29] = 2.30, *P* <.02) (Figure 1). In the PD-MCI group, 7 of 11 subjects fell into the Total Anosmia range while in the PD-NC, 5 of 21 were so classified.

**Table 2.** Cognitive Test Scores for PD-MCI and PD-NC Groups.

Cognitive Task	PD-MCI mean (SD)	PD-NC mean (SD)	Student's t	df	*P*	Cohen's d effect size
Block design	10.3 (2.8)	12.0 (2.8)	1.56	27	.130	0.609
CVLT (single trial)	−2.0 (0.7)	−0.7 (1.0)	4.1	31	< .001*	1.483
CVLT (total)	39.7 (10.0)	48.2 (8.5)	2.60	31	.013*	0.958
SDMT	−1.9 (0.6)	−0.6 (0.8)	4.50	30	.000*	1.800
VF letters	10.3 (3.5)	12.5 (3.0)	1.77	27	.087	0.693
VF categories	10.3 (3.2)	11.5 (2.9)	1.06	27	.301	0.412
TMT numbers	7.4 (3.1)	10.4 (1.9)	3.49	31	.001*	1.264
TMT switching	7.9 (3.4)	11.0 (2.1)	3.27	31	.003*	1.182
TMT motor	8.3 (3.1)	10.7 (1.6)	3.03	31	.005*	1.095
TMT scanning	7.0 (4.1)	9.8 (2.3)	2.52	31	.017*	0.913
Spatial span	10.7 (2.8)	11.6 (2.6)	0.84	27	.407	0.329

Note. Block design, VF letters, D-KEFS VF categories, D-KEFS. TMT numbers, TMT switching, TMT motor, TMT scanning, and spatial span are scaled scores (*M* = 10, *SD* = 3). CVLT (single trial) and SDMT are Z-scores (*M* = 0, *SD* = 1). CVLT (total) is a T-score (*M* = 50, *SD* = 10).

Abbreviations: CVLT, the California Verbal Learning Test; SDMT, the Symbol Digit Modalities Test; TMT, Trail Making Test; VF, Verbal Fluency.

*p <.05.

**Figure 2.**
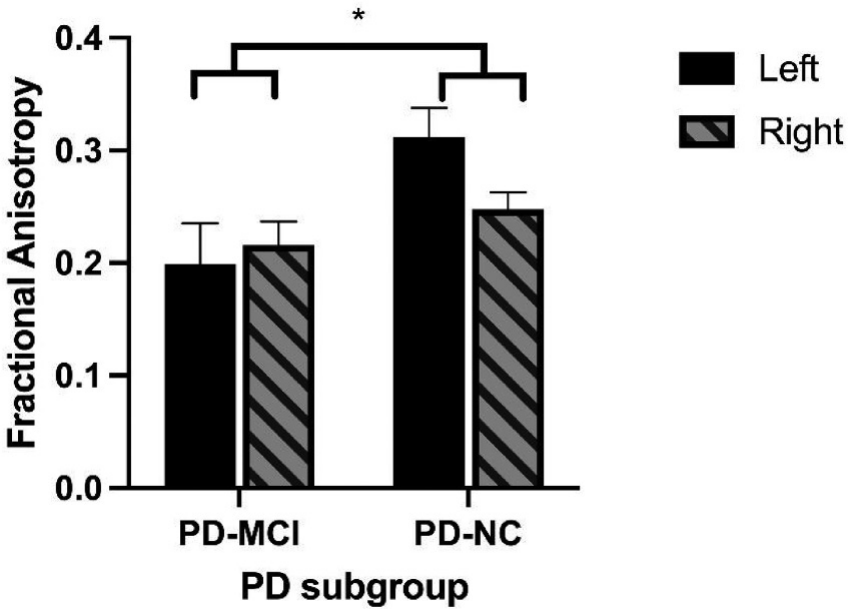
Mean left and right FA scores of PD-MCI and PD-NC groups. PD-MCI patients had significantly lower FA values than did the PD-NC. *P <.05.

Abbreviations: FA, fractional anisotropy; PD-MCI, Parkinson's disease with mild cognitive impairment; PD-NC, Parkinson's disease with normal cognition.

**Figure 3.**
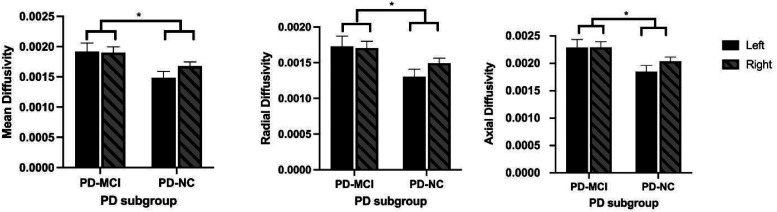
Left and right, Mean diffusivity, radial diffusivity, and axial diffusivity scores across PD-MCI and PD-NC groups. In all cases, PD-MCI patients had higher mean DTI values (mean diffusivity, radial diffusivity, and axial diffusivity) than the PD-NC patients.

Abbreviations: DTI, diffusion tensor imaging; PD-MCI, Parkinson's disease with mild cognitive impairment; PD-NC, Parkinson's disease with normal cognition.

Four subjects were excluded from the DTI analysis because their imaging followed different protocols and the data obtained were not comparable (2 in each of the 2 groups; no overlap with those who were missing UPSIT data). For all DTI parameters, the same pattern of findings was observed: there were no significant main effects for side, nor were there significant interactions between group and side. There were, however, group differences on all DTI parameters. On FA, PD-MCI had significantly higher values than PD-NC (F[1,27] = 5.8, *P* < .03; see Figure 1) and significantly lower values for MD, AS and RD (AD: F[1,27] = 6.8, *p* < .02; RD: F[1,27] = 6.1, *p* < .02; MD: F[1,27] = 6.4, *p* < .02; See Figures 2 and 3).

## Discussion

PD is a life altering neurodegenerative disorder that has widespread effects involving motor and non-motor symptoms. When combined with cognitive impairment the burden of PD is accentuated.^31^ The purpose of the current study was to identify 2 groups of people with PD—those with MCI and those with NC—and compare these 2 groups on olfactory functioning and DTI metrics. Our data suggests that the PD-MCI patients had poorer olfactory functioning and more microstructural abnormalities than did the people with PD-NC despite no differences in age, duration of illness and severity of motor symptoms.

As we anticipated, comparisons of cognitive test scores between PD-MCI and PD-NC groups demonstrated a broadly cognitively impaired group among PD patients. The PD-MCI group demonstrated impairments in multiple domains, including deficits in complex attention, information processing speed, working memory, new learning, and executive abilities.

The findings from olfactory function testing show that individuals with PD-MCI had significantly lower mean olfaction identification scores on the UPSIT than individuals with PD-NC. The PD-MCI subjects in our study fall into the “anosmia” category, while the PD-NC comparison group subjects fall into the “severe microsmia” category; thus, the latter group is still impaired relative to an age appropriate, healthy population. The finding of olfactory dysfunction being linked to worse cognitive function is consistent with the results of earlier studies that described links between the decline in cognitive and olfactory functions in PD patients.^15,32^ This evidence implies that the severity of olfactory dysfunction is related to cognitive decline and suggests that patients meeting the anosmia criteria may be at higher risk of eventually developing dementia and should, perhaps, be monitored more closely.

There were significant group differences for all DTI parameters measured in the olfactory system, including lower mean FA scores in PD-MCI patients relative to PD-NC controls. FA is sensitive to microstructural changes in white matter but is not indicative of the type of disturbance, although the lower scores in those with cognitive decline suggests the possibility of demyelination and disruption of FA by the presence of cerebrospinal fluid.^33^ Increased MD, AD, and RD values in the PD-MCI group (Figures 2 and 3) reflect less directionality of water flow in white matter, which suggests the possibility of increased edema or inflammation relative to the PD-NC group^34^ or increased axonal degeneration and/or decreased axonal density.^33^

In the larger study that included these patients, we showed that PD patients have ODs and show increased MD and decreased FA in the olfactory bulbs and tracts relative to healthy controls.^16-17^ It may be, therefore, that the PD-NC patients in this study had changes in DTI parameters relative to healthy controls. However, those with early cognitive impairment showed more extreme changes in DTI measures as well as in olfactory function than those that were cognitively more intact.

While white matter integrity of the olfactory bulbs and tracts in cognitively impaired PD individuals has not previously been explored, changes in microstructural integrity have been observed across many different brain regions in PD-MCI patients relative to control subjects.^35^ However, only few white matter tracts differentiate cognitively impaired from cognitively intact PD patients.^35^ Nevertheless, MD and FA alterations of frontal and corpus callosum white matter have been shown to correlate with the degree of cognitive impairment in PD patients.^36-37^ Grey matter changes are less likely to be found in PD-MCI relative to patients who are cognitively intact,^38-39^ perhaps as a result of white matter abnormalities preceding the appearance of grey matter changes.^36,40^

The DTI findings in the current study may be related to the ODs observed and could imply more rapid or extreme neurodegenerative changes affecting both olfactory and cognitive functions in this subset of patients. The combination of extreme changes in olfactory function with DTI changes in the olfactory system may identify a group of PD patients who are at higher risk of eventually progressing from MCI to dementia. Given that patients with early PD-MCI, whose cognition normalizes with disease progression, are also at risk of developing PDD, cognitive dysfunction early in the course of illness may be a harbinger of poorer outcome. The presence of anosmia, which can be detected with a simple test, should raise suspicion of both current mild cognitive impairments and of increased risk for future progression of cognitive decline.

This approach may be the first step towards identifying those who will consequently develop PDD. If early in the course of PD an individual presents signs of “anosmia”, it may be more likely that he/she is going to also present with cognitive impairment, compared to PD individuals whose olfactory scores are more moderately impaired. Poor olfactory function, early in PD raises suspicion, and more thorough clinical follow up might be warranted.

### Limitations and Future Directions

One limitation of this study concerns the use of the UPSIT. This test is widely used in research because of its simplicity and cost-effectiveness. Despite being a standard used for detecting OD, it is, however, susceptible to error and bias. The odorants used in the UPSIT may be more familiar to some individuals and therefore more easily named. The test has been criticized for having an American cultural bias,^41-42^ which could further limit its general usefulness. One alternative measure of OD involves the use of olfactory event-related potentials (OERPs)^43^ and has been used to examine changes in olfaction as a result of stimulation of the olfactory system.^12^ The use of OERPs, however, requires both additional equipment and expertise relative to the UPSIT.

A second limitation of this investigation was the small sample size of 33 patients. Despite the large effect sizes observed for findings related to both UPSIT scores and DTI measures (Table 2), studies using larger samples would increase confidence in the findings of this study. The small sample size may also have contributed to not detecting any significant differences in age or duration of illness since diagnosis (Table 1). A larger sample might reveal that such differences exist and that the olfactory and DTI differences observed are proxies for differences in the severity or stage of illness.

## Conclusion

The results of this study supported the hypothesis that PD patients with mild cognitive impairment would show more severe deficits in olfactory function and larger changes in olfactory system DTI measures than cognitively intact patients at a similar stage of illness. If confirmed in larger samples of patients, these results would suggest that it would be useful to screen PD patients for severe olfactory function deficits at early disease stages in order to identify those at higher risk of eventual progression to dementia.

## Notes

a. For the Symbol Digit Modalities Test, scaled scores were computed based on data collected from the normal control subjects participating in a larger imaging project, of which this study is part.
